# Executive function in systemic arterial hypertension: A systematic review

**DOI:** 10.1590/1980-57642018dn13-030004

**Published:** 2019

**Authors:** Natália Cristina Moraes, Ivan Aprahamian, Mônica Sanches Yassuda

**Affiliations:** 1 University of São Paulo Department of Neurology SP Brazil Department of Neurology, University of São Paulo, SP, Brazil.; 2 Faculty of Medicine of Jundiaí Department of Internal Medicine Division of Geriatrics and Gerontology SP Brazil Division of Geriatrics and Gerontology, Department of Internal Medicine, Faculty of Medicine of Jundiaí, SP, Brazil.

**Keywords:** hypertension, executive functions, cognition, systematic review, hipertensão, funções executivas, cognição, revisão sistemática

## Abstract

Systemic arterial hypertension (SAH) may be associated with worse cognitive performance, especially in tasks that evaluate the executive functions (EF).

**Objective:**

we aimed to review the evidence regarding which components of executive functions are most affected in adults with SAH.

**Methods:**

this systematic review used the PRISMA statement for searching Pubmed, Scielo and Lilacs databases with the keywords “executive function OR executive functioning AND hypertension”.

**Results:**

EF tasks were divided into shifting, inhibitory control and updating. A total of 9 cross-sectional and 3 longitudinal studies were selected. Only 3 studies did not report worse performance among SAH patients on EF tasks when compared to normotensive controls. The measures of shifting and inhibitory control were the most frequently investigated and reported as altered among SAH individuals, assessed mainly by the Stroop Test and Trail-Making Test part B, respectively.

**Conclusion:**

inhibitory control and shifting are the EF components most influenced by SAH. The results of this review may contribute to the devising of hypotheses about mechanisms underlying these cognitive impairments.

## RESUMO

A hipertensão arterial sistêmica (HAS) pode estar relacionada com pior desempenho cognitivo, principalmente em tarefas que avaliam as funções executivas (FE).

**Objetivo:**

este artigo teve como objetivo revisar as evidências sobre quais componentes das FE são mais afetadas em adultos com HAS.

**Metódos:**

esta revisão sistemática utilizou a metodologia PRISMA para buscas nas bases de dados Pubmed, Scielo e Lilacs com as palavras chaves “função executivas OU funcionamento executivo E hipertensão”.

**Resultados:**

as tarefas foram divididas em alternância, controle inibitório e atualização. Foram selecionados 9 estudos transversais e 3 longitudinais. Apenas 3 estudos não relataram pior desempenho entre os pacientes com HAS em tarefa de FE quando comparados aos controles normotensos. As medidas de alternância e controle inibitório foram as mais frequentemente investigadas e relatadas como alteradas entre os indivíduos com HAS, avaliadas principalmente pelo Stroop Test e Teste de Trilhas parte B, respectivamente.

**Conclusão:**

o controle inibitório e a alternância são os componentes da FE mais influenciados pela HAS. Os resultados desta revisão podem contribuir para a elaboração de hipóteses sobre mecanismos subjacentes a esses comprometimentos cognitivos.

Systemic arterial hypertension (SAH) is one of the most prevalent chronic diseases among adults, affecting around 600 million people in the Brazilian population.^1^ There are an estimated 7.1 million deaths annually from complications of this disease.^1^ Untreated SAH has negative consequences, such as the development of serious cardiovascular complications, including chronic renal failure, coronary artery disease (CAD), and stroke.^2^^-^4 More recently, researchers have examined the impact of SAH on cognition, especially on tasks associated with the concept of executive functions (EF).

The mechanisms by which SAH affects cognition are still not fully understood. In a recent paper, researchers reported that hypertension might alter the structure and function of cerebral blood vessels, leading to possible ischemic damage in regions of the white matter involved in cognitive processing.^5^ Other studies have suggested that the combination of advanced age and SAH fosters multifaceted interactions in pathophysiological pathways, leading, for example, to small vessel disease, altered regulation of blood flow and presence of signs of hyperintensity in the white matter.^6^ In addition, the relationship of SAH with increased cerebral vascular resistance and with diffuse lesions and multiple lacunar infarcts in the white matter is recognized, especially in the subcortical region.^7^^,^^8^ It is estimated that these brain changes can have a marked negative impact on performance of tasks associated with EF.

The concept of EF involves multiple important cognitive processes in regulating thoughts and behaviors.^9^^-^11 The term has been used to describe a number of skills that include various processing components such as: interrupting automatic and impulsive responses, starting tasks, resisting distractions and irrelevant interferences, switching between tasks, planning and monitoring intentional actions.^9^^,^^11^^-^13 Some theoretical models consider inhibition (inhibitory control), updating (working memory) and shifting or cognitive flexibility as the main domains of EF.^11^^,^^14^^-^17

Inhibitory control (IC) refers to the ability to deliberately inhibit dominant, automatic and impulsive responses or behaviors when necessary.^11^^,^^18^ Updating is closely linked to working memory and is responsible for constantly replacing some information with another during task performance.^11^^,^^17^ Finally, shifting involves the ability of performance control among different tasks, requiring rules to be maintained or disengaged, as needed.^11^^,^^17^^,^^18^

Some examples of tasks that evaluate the three main domains of EF are given in Table 1. It is important to note that cognitive tasks, in general, measure multiple domains of human cognition, but there is one domain that is understood as the central focus of the test.

**Table 1:** Main neuropsychological tests that evaluate inhibitory control, updating and shifting components of EF.

EF component	Neuropsychological task	Cognitive demand
**Inhibitory control**	Stroop Test^19^	Names of colors (e.g. "green") printed in a different color (e.g. "red"), where it is necessary to inhibit the reading of the word to report the color of the ink.
	Go-no-go tasks^9^	This type of task requires the subject to inhibit one response in favor of another, such as pressing a button when a stimulus appears, and not pressing when a different stimulus appears.
**Updating**	Digit Span Backwards^20^	Requires the subject to repeat a sequence of numbers backwards. The numbers must be retained mentally and worked with simultaneously.
	Verbal Fluency^21^	The subject has to utter as many words as they can in the same semantic or phonemic order within sixty seconds.
	Reorder Items^9^	The subject is asked to reorder names of animals or numbers by increasing size.
**Shifting**	Trail-Making Test part B^22^	The subject needs to connect numbers and letters, alternating them with numbers in ascending order and letters in alphabetical order as fast as they can.
	Wisconsin Card Sorting Task^23^	Each card can be classified by color, shape or number. The subject must deduce the classification criteria based on feedback provided by the examiner and shifting the classification rules whenever needed.

**Adapted from Miyake et al., 2000;^11^ Diamond, 2003;^9^ Rabinovici et al., 2015;^62^ Shao et al., 2014.^63^

Few studies have investigated the effect of SAH on different components of EF. Studies on the specific domains of EF affected by SAH may help identify the underlying mechanisms of impairment, given that components of EF may depend on different brain regions. For example, inhibitory control has been associated with frontal lobes, particularly the inferior frontal gyrus,^24^ while updating appears to recruit the basal ganglia.^25^ Shifting has been less examined in functional neuroimaging exams because it involves different parameters within the same task.^17^

Due to the scarcity of publications about the impact of SAH on the components of EF, the main objective of this study was to review the evidence regarding which components of executive functions are most affected in adults with SAH.

## METHODS

**Figure 1:**
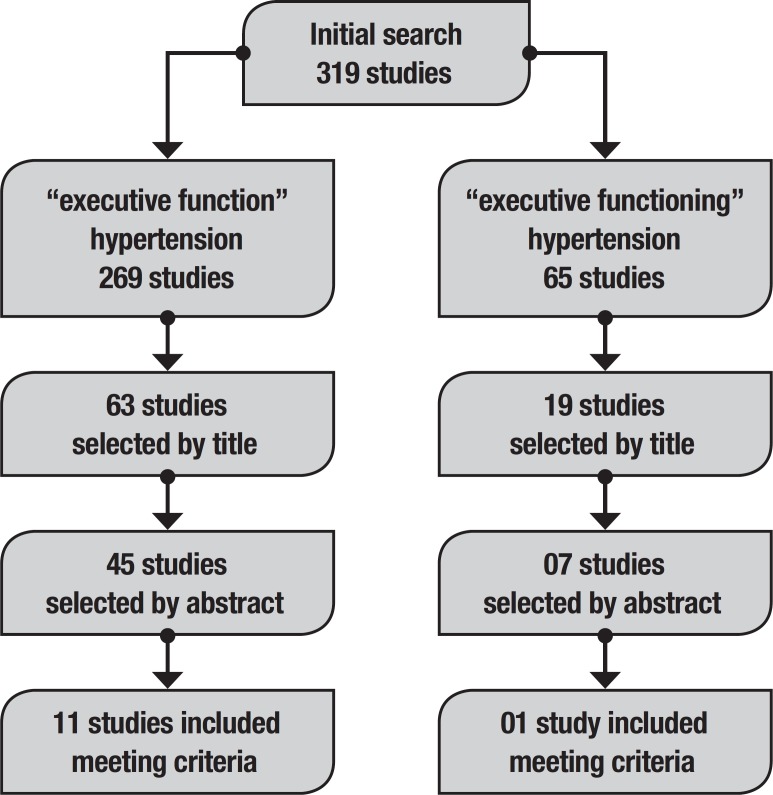
Literature search flow diagram.

This systematic review was performed according to the Preferred Reporting Items for Systematic Reviews and Meta-Analyses Statement (www.prisma-statement.org). The electronic databases Pubmed, Lilacs and Scielo were searched with the following keywords: “executive function OR executive functioning AND hypertension”. The searches were done between January 2017 and January 2019. A total of 319 studies were retrieved (Figure 1).

The titles and abstracts were analyzed and a selection of articles was made according to the following inclusion criteria: a) involve one or more cognitive measures, at least one of them being EF; b) be available in Portuguese or English; c) include hypertension data with objective measures and not only self-report; d) comprise studies involving young, middle-aged and elderly adults. Exclusion criteria were: a) investigations whose primary objective was other diseases - e.g. diabetes, dementia, obesity, dyslipidemia; b) intervention studies; c) research in participants with stroke; d) surveys that included participants with mild cognitive impairment (MCI), cognitive impairment without dementia (CIND) or dementia; e) studies that included hypertensive subjects not treated with medications; f) case series studies, case reports, editorials and reviews. This selection yielded a total of 50 articles.

Finally, the articles were read in full and discussed with another researcher regarding inclusion and exclusion criteria for the final selection of studies. Twelve articles were selected to be included in the review, respecting inclusion and exclusion criteria.

## RESULTS

There were 12 selected studies that investigated the relationship between SAH and EF, comprising nine cross-sectional and three longitudinal studies. Table 2 shows the studies characteristics, including sample profile, years of education, gender distribution, time with SAH diagnosis, follow-up time for longitudinal studies and countries of data collection.

**Table 2:** Main characteristics of selected studies.

Study	Country	Sample	Education (years)	Gender distribution	Diagnosis time	Follow-up time
**Cross-sectional**						
Waldstein et al., 1996^26^	USA	Hypertensive and normotensive young and middle-aged	9-21 years	Only men	N/A	
Kuo et al., 2004^27^	USA	Normotensive and hypertensive	12-20 years	44.3% women	N/A	
Vicario et al., 2005^28^	Argentina	Normotensive and hypertensive	4-11 years	62.2% women	5-30 years	
Hannesdottir et al., 2009^29^	England	Treated and untreated hypertensive and normotensive	<8 and >12 years	56.25% men	6 months-33 years	
Bucur & Maden, 2010^30^	EUA	Normotensive and hypertensive	12-19 years	52.9% women	N/A	
Giordano et al., 2012^31^	Italy	Normotensive and hypertensive	2-18 years	57% women	N/A	
Matoso et al., 2013^32^	Brazil	Normotensive and hypertensive	7-12 years	53% women	N/A	
Alipour et al., 2015^33^	Iran	Normotensive and hypertensive	3-10 years	54% women	N/A	
Li et al., 2015^34^	China	Normotensive and hypertensive	7-15 years	61.7% women	N/A	
**Longitudinal**						
Yasar et al., 2011^35^	USA	Normotensive and hypertensive stages I and II	<12 and >12 years	N/A	N/A	9 years
Vicario et al., 2011^35^	Argentina	Hypertensive	4-11 years	65% women	N/A	6 years
Chen et al., 2015^37^	Australia	Normotensive, pre-hypertensive and hypertensive	9-14 years	Only women	N/A	10 years

N/A: not applicable.

Tables 3 and 4 show cross-sectional and longitudinal findings for the associations between SAH and EF, respectively. In these tables, information on EF components was included, according to the model described by Miyake et al. (2000) and Diamond (2013).

**Table 3:** Cross-sectional studies on EF and SAH.

Authors, year of publication	Objective	Sample	EF tasks	Main results
Waldstein et al., 1996^26^	To investigate the interaction between age and hypertension on performance on neuropsychological tests. In this study, pharmacological treatment was discontinued two weeks before cognitive evaluation.	N=173; the groups were divided into young (≤40 years) and middle-aged (≥40 years), hypertensive and normotensive, totaling 4 groups.	Digit Span of the WAIS-II Scale, Phonemic Verbal Fluency, TMT part B and Stroop Test.	The young hypertensive group showed a significantly lower performance than the normotensive on the Stroop test, demonstrating worse inhibitory control. There was no difference between the middle age groups.
Kuo et al., 2004^27^	To determine the relationship between BP and different cognitive domains, and to analyze the relationship between decreased orthostatic pressure and cognitive functions in a relatively healthy elderly group.	N=70; ≥65 years old, elderly with stable chronic hypertension.	TMT part B and Verbal Fluency (Phonemic and Semantic).	Higher BP was associated with impairment in shifting performance as measured by TMT part B. Every 10 mmHg increase in systolic blood pressure (SBP) was associated with a 2.31 fold increase in the risk of impairment on this test.
Vicario et al., 2005^28^	To identify the effect of SAH on cognition and to differentiate this effect from other deterioration processes expected in aging.	N=90. There were 60 hypertensive and 30 normotensive aged ≥65 years.	Verbal and visual versions of TMT part B and Stroop Test.	The hypertensive subjects had more deficits on TMT part B, measure of shifting, with greater number of errors. The same group also exhibited deficits in IC performance, as measured by the Stroop Test.
Hannesdottir et al., 2009^29^	The hypertensive subjects had more deficits on TMT part B, measure of shifting, with greater number of errors. The same group also revealed deficits in IC performance, as measured by the Stroop Test.	N=80; ≥59 years, 40 subjects treated, 10 untreated and 30 controls (normotensive).	Digit Span of WAIS III Scale, TMT part B and Verbal Fluency (Phonemic and Semantic).	Compared with the normotensive subjects, the untreated hypertensive group had worse performance on the Verbal Phonemic Fluency test, used for evaluating updating and IC.
Bucur & Madden, 2010^30^	To develop a more precise definition of the cognitive differences between hypertensive and normotensive.	N=131, 71 normotensive and 58 hypertensive individuals, categorized into young (19-39 years), middle age (41-58 years) and elderly (60-79 years).	Stroop Test, TMT part B and Phonemic Verbal Fluency.	The group of older hypertensive patients had worse performance than normotensive patients of the same age group on all EF tests, indicating impairment of IC, updating and shifting.
Giordano et al., 2012^31^	To investigate the relationship of different components of BP with cognitive functioning and cognitive reserve in a sample and identify which domains are most affected.	N=288, of which 234 were hypertensive and 54 normotensive aged ≥50 years.	TMT part B and Phonemic Verbal Fluency.	There was no difference between the hypertensive and normotensive groups in relation to the EF test performance.
Matoso et al., 2013^32^	To compare the cognitive performance of hypertensive and normotensive elderly with at least 5 years of education.	N=45, 17 normotensive and 28 hypertensive patients aged ≥60 years and <80 years.	TMT part B.	Hypertensive patients were significantly slower than normotensive patients. There was also a difference in the performance of the hypertensive group on TMT part B when compared to the normotensive group, indicating shifting impairment.
Alipour and Goldust, 2015^33^	To assess the relationship between BP measurements (SBP, DBP and PP) and cognitive functions and cognitive reserve.	N=500, with 248 hypertensive and 252 normotensive patients, aged ≥62 years.	TMT part B and Phonemic Verbal Fluency.	There was no significant difference in EF measurements between the groups.
Li et al., 2015^34^	To evaluate the cognitive performance of hypertensive individuals in a large sample of ethnic Han people (China).	N=1007, of which 405 were hypertensive and 602 normotensive, aged 57-70 years.	TMT part B and Stroop test.	The hypertensive subjects had worse performance on the tests that evaluated IC and shifting when compared with normotensive controls.

N: sample size; WAIS III: Wechsler Adult Intelligence Scale Third Edition; TMT: Trail-Making Test; BP: Blood pressure; SBP: Systolic blood pressure; DBP: Diastolic blood pressure; PP: Pulse pressure; SAH: Systemic Arterial Hypertension; EF: Executive functions; IC: inhibitory control.

**Table 4:** Longitudinal studies on EF and SAH.

Authors, year of publication	Objective	Sample	EF tasks	Main results
Yasar et al., 2011^35^	To evaluate SBP or PP and cognitive impairment of elderly women over 9 years.	N=336, 103 normotensive, 124 hypertensive patients with SBP=140-159 mmHg and 109 hypertensive patients with SBP values ≥160 mmHg, aged 70-80 years.	TMT part B.	The HTN II had a five-fold increased risk of impairment on TMT part B, a measure of shifting, when compared to the normotensive group.
Vicario et al., 2011^36^	To observe the cognitive evolution of hypertensive patients over 6 years.	N=60, hypertensive patients aged ≥68 years.	TMT part B and Stroop Test.	At 6 years of follow-up, there was decline in shifting (TMT part B) and inhibitory control (Stroop test).
Chen et al., 2015^37^	To examine the associations between BP levels and cognition in middle-aged and older women over 10 years.	N=247; the groups were divided into normotensive (91), pre-hypertensive (108) and hypertensive (48), divided into middle-aged and older women.	TMT part B.	There was no significant difference between the groups, both in the initial and follow-up analyses.

N: sample size; WAIS III: Wechsler Adult Intelligence Scale Third Edition; TMT: Trail Making Test; BP: Blood pressure; SBP: Systolic blood pressure; DBP: Diastolic blood pressure; PP: Pulse pressure; SAH: Systemic arterial hypertension; EF: Executive functions.

In cross-sectional studies, the TMT part B test was used for all studies, but not all of them had altered results. The Stroop Test was also used by most of the studies. These two tasks were the most affected in hypertensive subjects, suggesting that IC and shifting are the most affected domains in hypertensive subjects. Only two studies found no impairment of EF in hypertensive individuals.^31^^,^^33^

In longitudinal studies, the results were more heterogeneous. Two studies found significant differences in the cognitive trajectories over time of normotensive and hypertensive individuals in EF components^35^^,^^36^ and one found no differences.^37^ Follow-up time, sample size, age range, and subject´s education may account for the differences in the findings of these studies. Tables 5 and 6 summarize findings from cross-sectional and longitudinal studies, respectively, indicating the tasks most affected in the hypertensive groups.

**Table 5:** Summary of impaired tests among patients with SAH in cross-sectional studies.

Studies	DF	PVF	SVF	Stroop Test	TMT B
Waldestein et al., 1996^26^	0	0	0	X	0
Kuo et al., 2004^27^		0	0		X
Vicario et al., 2005^28^				X	X
Hannesdottis et al., 2009^29^	0	X			X
Bucur and Madden, 2010^30^		X		X	X
Giordano et al., 2012^31^		0			0
Matoso et al., 2013^32^					X
Alipour & Goldust, 2015^33^		0		0	0
Li et al., 2015^34^				X	X

0: applied, but not impaired; X: impaired; DF: Digit Span Backwards; PVF: Phonemic Verbal Fluency; SVF: Semantic Verbal Fluency; TMT B: Trail-Making Test part B.

**Table 6:** Summary of impaired tests among patients with SAH in longitudinal studies.

Studies	DF	PVF	SVF	Stroop Test	TMTB
Yasar et al., 2011^35^					X
Vicario et al., 2011^36^				X	X
Chen et al., 2015^37^					0

0: applied; X: changed; DF: Digit Span Backwards; PVF: Phonemic Verbal Fluency; SVF: Semantic Verbal Fluency; TMTB: Trail-Making Test part B.

Table 5 shows that the studies of Alipour and Goldust (2015)^33^ and Vicario et al. (2005)^28^ used the same tasks to analyze shifting (TMT part B) and IC (Stroop Test), but had discordant results. Alipour and Goldust (2015)^33^ evaluated 500 individuals with a mean age of 76.14±12.65 years using the MMSE (Mini-Mental State Examination), Digit Span, story recall, CLOX Test (Clock Drawing Test), TMT parts A and B, and the Phonemic Verbal Fluency test. The hypertensive group demonstrated significantly lower performance on the MMSE, story recall and the CLOX.

Giordano et al. (2012)^31^ investigated the relationship of the different components of BP (PP, DBP and SBP) with cognitive function in a sample of 288 subjects with a mean age of 73.5±10.1 years. The tests applied were the MMSE, Digit Span, story recall; TMT part B, Phonemic Verbal Fluency, CLOX, TMT part A and one test of overlapping figures. The hypertensive group had a significantly poorer performance on the MMSE, the immediate and delayed story recall, and the CLOX. SBP and PP were independent predictors of cognitive impairment.

The other six studies found significant differences between hypertensive and normotensive groups. Waldstein et al. (1996)^26^ examined the interaction between age and SAH as predictors of cognitive performance in a sample of 123 hypertensive patients, compared to 50 normotensive subjects. The protocol consisted of Ryan's Verbal Paired-Associative Learning Test, Logical Memory, Digit Span and Digit Symbol, Recurring Word test and Recurring Faces test, TMT parts A and B, Phonemic Verbal Fluency with initials F, A and S, Grooved Pegboard test, Finger Tapping Hand Test, Hand Dynamometer test, Maze Learning Test, Corsi Blocks, and the Stroop test. Young hypertensive participants had significantly lower performance on the Stroop Test, while the hypertensive and normotensive middle-aged subjects did not differ on this test. The young and middle-aged hypertensive participants presented more deficits than the normotensive subjects on the motor dexterity tests.

Kuo et al. (2004)^27^ analyzed the relationship between orthostatic BP decline and cognitive functions in a group of 70 elderly individuals aged 72.0±4.4 years with the Logical Memory, Visual Reproduction, TMT parts A and B and Verbal Fluency tests. Elevated BP was associated with impairment on the TMT part B where, for every 10 mmHg increase in SBP, there was a 2.31 fold increase in the risk of TMT part B impairment. The increase in SBP was not associated with the other measures.

Vicario et al. (2005)^28^ evaluated 60 hypertensive patients from a cardiology clinic with mean age of 71.5±4.2 years using the MMSE, TMT parts A and B, the New York University Paragraph Test, Stroop Test and Verbal Phonemic Fluency, and the alternating series test of drawing copy. The hypertensive group had lower performance on the paragraph recall, TMT part B and Stroop test.

Hannesdottir et al. (2009)^29^ evaluated the MMSE, a short form story of WASI, Digits of WAIS III Scale, Phonemic Verbal Fluency, TMT parts A and B, Logical Memory I and II among treated and untreated hypertensive subjects and healthy controls. Treated hypertensives performed significantly worse than controls on the immediate and late recall of the Logical Memory tests. Untreated hypertensive participants scored lower on the MMSE, Verbal Phonemic Fluency and TMT A and B, compared to controls.

Bucur and Madden (2010)^30^ recruited 134 hypertensive and normotensive individuals, evaluating them with the phonemic verbal fluency, TMT A and B, Stroop Test and a computerized version of the Symbol Digit. The z-scores of EF of the hypertensive group of older adults were significantly lower than those of the hypertensive younger adults.

Matoso et al. (2013)^32^ selected 45 participants, 17 normotensives and 28 hypertensives. The neuropsychological assessment consisted of the Cambridge Cognition-Revised (CAMCOG-R), TMT parts A and B, and the Auditory-Verbal Learning (RAVLT). The hypertensive group had lower performances on the CAMCOG-R, TMT A and B, and RAVLT total score.

Li et al. (2015)^34^ evaluated the cognitive performance of Chinese hypertensive individuals. Cognition was evaluated by the MMSE, RAVLT, Rey's complex figure, the ROCF copy test, TDR, Symbol Digit, Verbal Fluency, the Boston Naming Test, Stroop test, and TMT parts A and B. Hypertensive subjects had lower performance on memory tests (RAVLT), EF (Stroop and TMT part B) and MMSE compared to controls.

Yasar et al. (2011)^35^ examined whether elevated SBP or PP in elderly women was associated with changes in cognitive abilities at baseline and over a period of 9 years. The cognitive battery consisted of the MMSE, TMT parts A and B, immediate and late recall of the Hopkins Verbal Learning Test-Revised (HVLT-R). In the cross-sectional analyses, the subjects of the HTN 1 group (SBP=140-159 mmHg) had a lower chance of impairment on the TMT part A compared to the control group. In the longitudinal analyses, the risk of cognitive impairment on any given test did not differ by SBP level. When participants were stratified according to age, subjects aged 76 to 80 years of the HTN 2 group (SBP ≥160 mmHg) had a five-fold increased risk of impairment on the TMT part B compared to the control group.

The study of Vicario et al. (2011),^36^ studying cognitive impairment in a cohort of hypertensive individuals, identifying affected domains and correlating cognitive performance with the measures of BP, recruited hypertensive subjects who were diagnosed at the age of 65 and followed up for six years. The neuropsychological evaluation was composed of the MMSE, New York University Paragraph Test to evaluate short and long-term episodic memory; TMT parts A and B, the CLOX and Stroop Test. The tests were administered at baseline and every 2 years of follow-up. Throughout the follow-up, short and long-term memory remained unchanged. However, there was a marked decline on the Stroop test and the TMT part B, with significant decline after the fourth year of follow-up.

Chen et al. (2015)^37^ examined the relationship between BP and cognition in middle-aged and elderly women. The study was based on data from 247 participants, whose BP was measured between 1992 and 2002. Cognitive assessment included the TMT part B, Symbol Digit Modalities Oral Test Version (SDMT), while Verbal Episodic Memory was evaluated using the Australian-modified California Verbal Learning Test (CVLT) and a 10-item supraspan word-list task. Analyses showed that normotensive women had better memory than those with pre-hypertension or hypertension. At follow-up, there was a significant difference between the two hypertensive groups in immediate episodic memory.

## DISCUSSION

The objective of this review was to systematize research evidence about the possible impact of SAH on aspects of EF. Twelve articles were analyzed and, in most studies, shifting and IC proved to be the components of EF functioning that are impaired among hypertensive patients, usually measured by the TMT part B and the Stroop Test, respectively. Of the studies reviewed, 11 evaluated shifting (6 indicated impairment among hypertensive patients), 6 evaluated IC (5 indicated impairment) and 6 evaluated updating (2 indicated impairment). Thus, updating was the EF aspect with the least impairment.

Among the factors that may explain this result is the recruitment of the fronto-parietal network by several EF tasks.^38^^,^^39^ The activity of the frontal network is closely related to cerebral blood flow,^40^ and may be influenced by alterations in the vasculature present in hypertension which would contribute to executive dysfunction.^41^^,^^42^ Some studies^43^^,^^44^ have shown that the prefrontal region atrophies more readily in hypertensive subjects when compared to other regions of the brain and to the brain of normotensive individuals, suggesting that hypertension particularly impairs EF.

In addition to evaluating the cognitive performance of hypertensive patients, Li et al. (2015)^34^ investigated the neural mechanisms involved using functional magnetic resonance imaging and diffusion tensor imaging. Hypertensive patients had poorer performance in shifting and IC tasks than normotensive subjects besides abnormal patterns of activation in the left fronto-parietal network. Some studies have reported that these regions are more susceptible to the deleterious effects of hypertension,^39^^,^^45^ as there may be regional reduction of cerebral blood flow in this condition.^45^^,^^46^

In the cross-sectional study by Goldstein et al. (2013),^47^ about 50% of hypertensive patients were unable to complete the TMT part B, in addition to having more errors than normotensive subjects. Similar results were found for performance on the Stroop test. In the longitudinal study by Vicario et al. (2011),^36^ hypertensive patients showed progressive impairment in these two tests during follow-up. These data suggest that shifting and IC tests may indicate continued impairment in EF due to prolonged exposure to SAH.

It is known that cardiovascular diseases can alter cerebral blood flow, leading to microinfarction in the frontal lobes, with implications for EF.^27^ SAH has been recognized as representing a risk for the development of cerebrovascular diseases, which may be accompanied by macro and micro cerebral lesions.^48^ Two cohort studies indicated that SAH is an important risk factor for micro infarcts.^49^^,^^50^ The association between BP and micro infarcts was observed in subjects younger than 80 years old, and was restricted to higher SBP, but not DBP.^49^

The IC and shifting measures have been highlighted in this review as the most altered aspects of EF, whereas in the studies that evaluated updating, only two^29^^,^^30^ found impairment in this component among hypertensive patients. Updating, usually measured by Digit Span Backwards and Verbal Fluency, requires replacement and maintenance of information in working memory, as opposed to shifting tasks in which information is constantly updated (without partial maintenance).^25^ It is possible that specific working memory factors involved in updating (temporary data maintenance) tasks may require less executive processing and thus would be less vulnerable to the effects of SAH.^17^

Many EF tasks require monitoring of information relevant to the task objective and inhibiting irrelevant responses.^51^^,^^52^ For this reason, IC impacts the ability to maintain and manage goals and can influence the processing in progress on several tasks.^53^^-^55 This hypothesis could explain why five out of the six studies that included IC measurements indicated impaired results, pointing to poorer performance among hypertensive patients, as IC may represent a central aspect of EF.

There is evidence that executive functioning is the most impaired cognitive aspect in hypertensive subjects.^55^^-^61 However, most of these studies focused on only one or two of the EF components. The studies identified by this review revealed that IC and shifting are the components of EF most frequently impaired among hypertensive participants. Future studies should include in their protocols neuropsychological tests that evaluate the three aspects of EF of hypertensive patients, correlating them with level of disease severity, time since diagnosis and treatment.

Future studies should investigate in more depth the possible relationships among the EF components and how these may change in the presence of diseases such as SAH. In addition, further studies should investigate the impact of SAH-associated impairment in EF components in everyday life, assessing subtle changes in activities of daily living among individuals with hypertension.
